# Malaria.

**DOI:** 10.3201/eid0707.017725

**Published:** 2001

**Authors:** A Teklehaimanot, G Keusch, S Binder

**Affiliations:** World Health Organization, Geneva, Switzerland.


### Malaria

**Awash Teklehaimanot,* Gerald Keusch,† and Sue Binder‡**
* World Health Organization, Geneva, Switzerland; †National Institutes of Health, Bethesda, Maryland, USA; and ‡Centers for Disease Control and Prevention, Atlanta, Georgia, USA

Malaria remains a major killer of young children and an enormous economic drain on developing countries. The purpose of this conference panel was to explore two major initiatives to build capacity for prevention and control of malaria.

### Roll Back Malaria

Awash Teklehaimanot, acting project manager for Roll Back Malaria (RBM), described its initiative. Each year, more than 300 million clinical cases of acute malarial illness occur, mainly affecting the world's poorest populations. More than 1 million people die each year from malaria, and 90% of these deaths occur in children in sub-Saharan Africa. Malaria is also a substantial impediment to human development in poor countries. It slows economic growth in Africa by up to 1.3% each year; the short-term benefits of malaria control have been estimated at U.S. $3 to $12 billion per year. Malaria is a growing concern as antimicrobial resistance against multiple drugs becomes more widespread and malaria develops in areas previously malaria-free.

The RBM partnership, launched by World Health Organization (WHO) Director-General Grö Harlem Brundtland in October 1998, is committed to cutting the global malaria burden in half by 2010. In Africa, where most malaria occurs, the RBM partnership builds on a history of malaria control and a political commitment to eliminating the disease, which has never been higher. For example, the African Heads of State Summit to Roll Back Malaria, held in Abuja, Nigeria, on April 25, 2000, marked the first meeting of African political leaders to discuss the human and economic consequences of malaria on their continent. At the summit, heads of several development agencies pledged $750 million in new money and discussed concrete action to be taken over the next decade.

The core elements of RBM strategy include 1) ensuring rapid diagnosis and early treatment within or near the home; 2) making insecticide-treated mosquito nets (ITNs) available and increasing access to other vector control measures, such as environmental management to control mosquitoes; 3) making pregnancy safer through preventive intermittent malaria treatment for pregnant women; 4) improving epidemic preparedness through improved surveillance and appropriate rapid response; and 5) supporting focused research to develop new medicines, vaccines, and insecticides.

To implement these core interventions on a large-scale, the RBM partnership recognizes the need to 1) strengthen the capacity of health systems and services; 2) work with and through other sectors such as education, public works, women's development, agriculture, and local government; 3) involve other groups, such as those in the private sector, and 4) sponsor focused applied research and development of effective tools and approaches. In addition, technical support networks comprised of experts with practical experience and from various institutions have been established to provide a link between universities, disease control operations, and international experts.

Some recent promising developments include ITNs with long-lasting insecticide; initiatives to create commercially sustainable markets for ITNs; more effective and less expensive antimalarial drug combinations; concerted efforts to reduce tariffs and taxes on antimalarial commodities, such as drugs and nets; and partnerships with other international health programs, such as the Integrated Management of Childhood Illness program, to both ensure more efficient health systems that address all diseases of poverty and to improve medical treatment of children. For further information about RBM, please visit their website at www.rbm.who.int.

### Multilateral Initiative on Malaria

Gerald Keusch described the Multilateral Initiative on Malaria (MIM) as an alliance of organizations and individuals working together to increase malaria research in Africa and to facilitate global collaboration, coordination, and capacity-building. MIM's roots can be traced back to 1995 when the National Institutes of Health (NIH) organized an initial planning meeting. This was followed in 1997 by an international conference in Dakar, Senegal, which was notable for the prominent role played by African malaria research scientists. After follow-up meetings in The Hague and in London, MIM was officially launched in late 1997, with the first secretariat housed at the Wellcome Trust. In 1999 the 1st International MIM Conference was held in Durban, South Africa, to bring the malaria research and control communities together. MIM's secretariat is intended to rotate among member organizations; since June 1999, it has been housed at the Fogarty International Center of NIH.

MIM has several objectives: 1) to raise international public awareness of the problem of malaria; 2) to promote global communication and cooperation on malaria; 3) to develop sustainable malaria research capacity in Africa; and 4) to ensure that research findings are applied to malaria treatment and control.

To date, MIM has had several notable accomplishments. With funding from NIH, the World Bank, the Rockefeller Foundation, WHO, and the governments of Norway, France, and Japan, MIM and WHO's Tropical Disease Research (TDR) program formed a MIM-TDR Research and Capacity-Building Grants Program. To date, 20 grants have been given through which $6 million has been distributed. The grants embody several of the guiding principles of MIM, such as an

Address for correspondence: Sue Binder, Centers for Disease Control and Prevention, 4770 Buford Highway, NE, Mailstop K02, Atlanta, GA 30341, USA; fax: 404-488-4422; e-mail: sbinder@cdc.gov emphasis on partnerships, decision-making by African scientists, and a strong scientific basis for the funded research. To support a variety of research programs, MIM has also developed the Malaria Research and Reference Reagent Resource Center, which provides high quality reagents and materials to investigators who are, or wish to be, involved in malaria research. NIH's National Library of Medicine has taken responsibility for enhancing the capacity of African scientists to do research by establishing and supporting access to communications and information resources. A number of research networks are online using very small aperture telecommunications (VSAT) technology for Internet access. This allows for shared databases, electronic mail and discussion groups, access to published literature, and use of remote sensing technologies. Information about the progress of MIM is shared through meetings, a newsletter, and on the internet at http://mim.nih.gov

Future goals of MIM include stabilizing funding for the MIM-TDR grant program, developing new partnerships, and creating new training opportunities, such as training on research management. Scientific research on *Plasmodium vivax* and on malaria-related anemia is being conducted. Interactions with RBM are well-established and coordinated. The 2nd International MIM Conference is scheduled for 2002 in Tanzania.

---

### Institutional Review Boards: Consideration in Developing Countries

**Jean William Pape**
Cornell University Medical College, New York, New York, USA, and Groupe Haitien d'Etude du Sarcome de Kaposi et des Infections Opportunistes, Port-au-Prince, Haiti

Institutional review boards (IRBs) play an essential role in protecting the rights of volunteers involved in research projects. Their function has become more complex, particularly concerning projects conducted in developing countries. But can IRBs in the United States guarantee the protection of human subjects involved in research projects in developing countries?

IRBs have no effective way of controlling what goes on in the field. The complex ethical clearance process does not determine whether persons engaged in research projects in developing countries are fully aware of the major aspects of the studies they participate in. The clearance process includes the IRB approval and consent forms. Required U.S. consent forms are too long and the language too complicated to be certain all participants have a full understanding of the study. The forms also appear to be intended more to offer legal protection to sponsoring agencies than to protect the welfare of the volunteer. Most importantly, the forms do not guarantee that volunteers have fully understood the objectives, risks, and benefits of the study and the extent of their voluntary participation. To protect volunteers as well as all persons and institutions involved, these forms must not only communicate necessary information concerning the study to be conducted but also evaluate volunteers' knowledge and their desire to participate. To achieve this goal, we propose to use a simple questionnaire administered by a team not involved in the volunteer recruitment process. We have used such a questionnaire to evaluate potential volunteers for a phase-II HIV vaccine trial. Although volunteers had three intensive, 2-hour counseling sessions, only half responded correctly to all 21 questions. The others were referred for additional counseling and reevaluation.

The IRB process requires that collaborative projects with U.S. institutions have clearance from multiple IRBs. Each IRB meets generally once a month and uses its own consent forms. Each has its own set of rules. Each will respond with different concerns that must be addressed. The approval process may create a lag time of 3 to 12 months to obtain ethical clearances for a project lasting 12 to 24 months.

The ethical clearance process can be simplified in several ways: 1) All studies supported by NIH should have a unique IRB application form and a unique IRB consent form. 2) A certain percentage of the research grant should be allocated to support the ethical clearance process. Ethical support should be available at the grant's initiation. 3) While waiting for the formal ethical clearance and final consent, potential volunteers could be counseled and evaluated. 4) The primary responsibility of local and national IRBs should be clearly determined. IRBs must share responsibilities to achieve the greatest benefit for volunteers. 5) A mechanism must be developed to resolve conflicts between IRBs from developed and developing countries. Yearly meetings of IRBs from host and sponsoring institutions should take place to facilitate the exchange of documents and other information.

---

Address for correspondence: Jean William Pape, Division of International Medicine and Infectious Diseases, Cornell University Medical College, Room A-421, 1300 York Avenue, New York, NY 10021 USA; fax: ; e-mail: jwpape@gheskio.org

